# Is it possible to implement a complex adaptive systems approach for marine systems? The experience of Italy and the Adriatic Sea

**DOI:** 10.1016/j.ocecoaman.2017.09.019

**Published:** 2017-11-15

**Authors:** Emanuele Bigagli

**Affiliations:** Wageningen University, Laboratory of Geo-Information Science and Remote Sensing, Droevendaalsesteeg 3, 6708 PB, Wageningen, The Netherlands

## Abstract

• This paper evaluates the implementation of the MSFD in the Adriatic Sea.
• The MSFD is the first policy for marine complex adaptive systems in the EU.
• Ecological and jurisdictional boundaries overlap and cross-border cooperation is low.
• Integrative assessments of marine systems may be impossible to achieve.
• Relative isolation of theoretical approaches and management practices.

## 1 Introduction

Oceans are fundamental components of global material and energy cycles (Costanza, 1999). They host critical, unique habitats for thousands of species of flora and fauna (Hattam et al., 2015), and provide essential services to humans, like food, transportation, leisure and recreation, waste dumping, as well as holding non-use values especially among indigenous communities (Barbier, 2012). Currently, anthropogenic and climate-related stressors challenge the health of nearly every part of the oceans (Halpern et al., 2008), altering physical, chemical and biological properties and impacting on their capacity to regulate globate climate and weather, on ocean productivity, and contributing to loss or degradation of marine habitats and biodiversity (IOC/UNESCO, 2011).

The failure of traditional, centralised, sector-based ocean management approaches (Berkes et al., 2003, Guerry, 2005, Curtin and Prellezo, 2010, Katsanevakis et al., 2011) pushed scientific research to develop conceptual and methodological frameworks based on the conceptualisation of coupled environmental and human systems as complex adaptive systems. Such approaches, which may be included within the broad label of the so-called Ecosystem Approach, or Ecosystem-Based Management (EBM) (for a review, see Arkema et al., 2006), focus explicitly on the recognition of humans as key elements of the ecosystem; they acknowledge the complex linkages between ecological and socio-economic components; and they introduce the need to improve management through systematic evaluation, while promoting shared responsibility across stakeholders and applying a precautionary approach (Grumbine, 1994, Arkema et al., 2006, De Jonge et al., 2012).

These principles started to be introduced in the texts of several international agreements (see e.g. the United Nations Convention on Biological Diversity and the Agenda 21 Chapter 17 on the protection and rational use of the oceans), as well as into management practices around the globe, especially in the USA (Ruckelshaus et al., 2008), Canada (Curtin and Prellezo, 2010) and Australia (Ruckelshaus et al., 2008, Curtin and Prellezo, 2010). In Europe, this process started in 2000 with the issue of the Water Framework Directive (WFD; 2000/60/EC), for the protection of freshwater systems, including transitional and coastal waters, and extended further offshore in 2008 with the Marine Strategy Framework Directive (MSFD; 2008/56/EC). The MSFD stands as a turning point of European marine governance, as it introduces for the first time in Europe a geographical, learning-based approach to the assessment and management of marine systems (Bigagli, 2015). This approach is based on three main features: the use of geographical criteria, instead of jurisdictional and administrative ones, to the definition of marine regions and sub-regions; the objective of clean, healthy and resilient oceans and seas, to be achieved through Good Environmental Status (GES) criteria and indicators; and the application of policy iteration and learning strategies (Bigagli, 2015).

The translation of a complex adaptive systems approach into the assessment and management of marine systems has been acknowledged as difficult and particularly challenging (Arkema et al., 2006, Levin et al., 2009, Tallis et al., 2010, Katsanevakis et al., 2011). Different approaches between scientists and managers may result in the missing of critical ecological and human factors into management plans (Arkema et al., 2006). Moreover, it may be difficult to translate complex ecological principles into clear and understandable management strategies (Levin et al., 2009). In addition to this, major science and knowledge gaps exist on critical complex components and dynamics (Katsanevakis et al., 2011).

The picture is further complicated in Europe. The MSFD was introduced into an already complex European governance landscape for the marine environment, characterised by a variety of maritime activities, often in conflict, and regulated by fragmented, sector-based public policies operating at multiple levels (De Jonge et al., 2006, De Jonge, 2007, Van Tatenhove, 2013, Boyes and Elliott, 2014). The MSFD tries to address this fragmentation, by requiring Member States to build coordination and cooperation mechanisms on two levels: among different policies, the most notable ones being the Water Framework Directive (WFD; 2000/60/EC) and the Common Fisheries Policy; and with other MS sharing the same marine region or sub-region.

However, previous research identified three major limitations in the text of the MSFD (Bigagli, 2015). They are related to: 1) the insufficient capacity of the MSFD to have a geographical, social-ecological approach to marine areas, including the limited capacity to coordinate among Member States (MS) sharing the same marine region or sub-region; 2) the limited capacity of the MSFD to include socio-economic aspects, and their interactions and outcomes with ecological systems; and 3) the limited strength of the requirements to coordinate with other laws and policies. The results of this investigation are summarised in the first column of Table 1.

**Table 1:** The three components of the framework for marine complex adaptive systems assessment and management; related results of previous research on the analysis of EU legislation (Bigagli, 2015) and the specific research questions for the case study.

Framework for marine complex adaptive systems (from Bigagli, 2017)	EU legislation strengths and weaknesses (from Bigagli, 2015)	Research questions for the case study of the Adriatic Sea
**Unit of management** Social-ecological system (AM), including connected socio-technical systems (TM)	The MSFD identifies marine regions and sub-regions following bio-geographical criteria Limitations: 1. The scope is limited to jurisdictional waters; high seas are excluded 2. Limited strength of the requirement to cooperation for Member States sharing the same marine region or sub-region 3. Limited strength of the requirement to coordinate with the spatial scopes of other existing laws and policies	1. Where is the MSFD implemented? 2. Are there in place mechanisms for cross-border cooperation among countries sharing the same marine region or sub-region? 3. Are there in place mechanisms for spatial coordination with other legislation?
**Objective of management** Achieve or maintain the ecological resilience (AM), in coordination with transitions of unsustainable socio-technical systems (TM)	The MSFD Good Environmental Status (GES) operationalises the concept of ecological resilience Limitations: 1. The GES characterisation overlooks socio-economic components, their internal dynamics and influences on ecological resilience 2. Limited strength of the requirement to coordinate GES objectives with other Member States sharing the same marine region or sub-region 3. Limited strength of the requirement to coordinate with the objectives of other legal acts, which may overlap and conflict with GES	1. How are GES identified and intended to be achieved? 2. Have objectives been set at Adriatic-level, or at the scale of other related SESs? 3. Have the objectives of other legislation been integrated with MSFD into a common framework?
**Structure of management** Iterative, learning-based policy cycle, based on thorough knowledge and understanding of the systems (AM and TM)	The MSFD requires an iterative, learning- and science-based policy cycle Limitations: 1. Lack of coordination with other laws and policies: different policy cycles with overlapping and misaligned phases and timelines of implementation	1. How is the MSFD policy cycle implemented? 2. Is the MSFD policy cycle coordinated at Adriatic scale? 3. Are there initiatives and mechanisms in place to foster the coordination of MSFD with other legal acts?

The implementation of a public policy is not a straightforward process, especially in the EU, with multiple decision points and opportunities for national governments to ‘erode’ the original objectives of a policy (Dimitrakopoulos and Richardson, 2001). For this reason, there is the need to evaluate whether the limitations identified on paper are translated (or not) into practice. The purpose of this paper is to present the result of such evaluation, through the investigation of the current management practices of the Adriatic Sea. Lessons will be also derived on the real possibility to implement a complex adaptive systems approach to the assessment and management of marine systems.

**Fig. 1:**
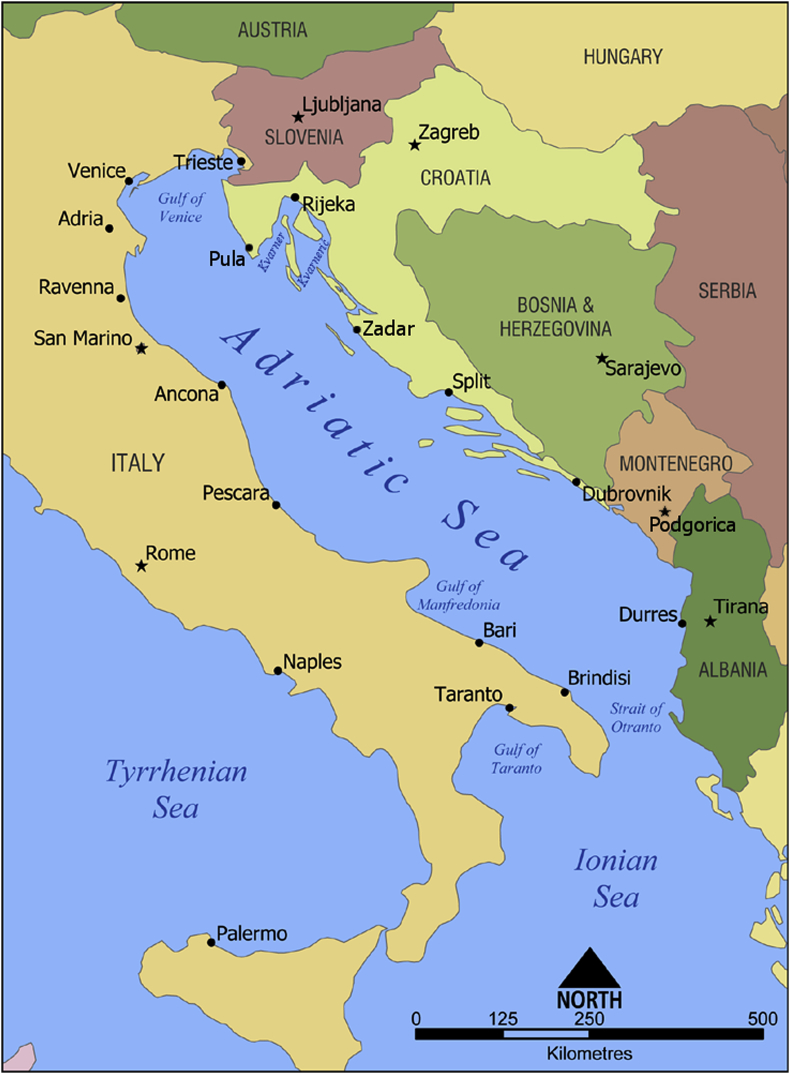
Map of the Adriatic Sea. Licensed under Creative Commons Attribution-Share Alike 3.0 via Wikimedia Commons - http://commons.wikimedia.org/wiki/File:Adriatic_Sea_map.png#mediaviewer/File:Adriatic_Sea_map.pngCC BY-SA 3.0.

The Adriatic Sea is a shallow, semi-enclosed basin located in the northern, central part of the Mediterranean Sea (see Fig. 1). For its bio-geographical characteristics, the MSFD identified it as a marine sub-region of the Mediterranean Sea. Moreover, the European Commission identified the Adriatic-Ionian area as one of the European macro-regions, and launched in 2014 a Strategy for the Adriatic and Ionian Region (EUSAIR, European Commission, 2014), aiming at creating synergies and fostering coordination among all territories in the area. In parallel, an increasing number of scientific, political and economic initiatives have been launched, aiming at protecting the marine environment and fostering sustainability and socio-economic integration among the EU (Croatia, Greece, Italy and Slovenia) and non-EU coastal countries (Albania, Bosnia-Herzegovina and Montenegro). An integrated, ecosystem-based approach is fundamental to preserve essential ecological processes and achieve or maintain the ecological resilience of the Adriatic Sea social-ecological system, while at the same time fostering the sustainability of maritime activities. For these reasons, it is important to verify the extent to which an integrated approach for the assessment and management of the Adriatic Sea social-ecological system is already in place, and identify major strengths and weaknesses in current practices.

The paper is structured as follows. Section 2 presents a framework of reference for marine complex adaptive systems and the methodology followed to develop the case study and derive the required information. Section 3 describes the case study area of the Adriatic Sea. Finally, section 4 presents the results of the investigation.

## 2 Methodology

### 2.1 A framework for marine complex adaptive systems

The case study investigation presented in this paper is based on the findings of previous research, where a framework for the assessment and management of marine complex adaptive systems was developed (Bigagli, 2017), and used to evaluate the current international and European legal frameworks for the management of marine systems (Bigagli, 2015, Bigagli, 2016). The framework is based on two promising conceptual and methodological frameworks to manage change and achieve sustainability of complex environmental and human systems (Foxon et al., 2009): Adaptive Management (AM) and Transition Management (TM). The starting point is that both AM and TM have important limitations. On the one side, AM practices tend to focus on ecological aspects, using frameworks for analysis and conceptualisation that may not fully consider the complexity of social systems and their dynamics (Binder et al., 2013). On the other side, TM strategies tend to be applied to specific areas or sectors in isolation, while at the same time not always considering environmental considerations into system assessment (Dryzek, 2013, Olsson et al., 2014, Pereira et al., 2015). The framework illustrated in Table 1 allows overcoming these limitations through the combination of both approaches, along three dimensions: the unit of management; the objectives of management; and the structure of management (see first column of Table 1).

More into detail, management of marine complex adaptive systems should clearly identify marine social-ecological systems and socio-technical systems, and assess the complex interactions and influences between socio-economic patterns of production and consumption, and ecological components. The objective of management should be to achieve or maintain marine ecological resilience, defined as the ability of a system to withstand shocks, maintain stability during disturbances and rebuild itself when required (Carpenter et al., 2001), in coordination with the transitions of unsustainable socio-technical systems, i.e. socio-economic systems that have a negative impact on the ecological resilience of connected social-ecological systems. Finally, iterative, learning-based approaches are required, where the knowledge of the system is at the basis of the development of a vision, policy options and management measures, whose effectiveness should be monitored in order to ‘learn’ from results and inform a new phase of vision and objectives setting. Such policy cycles should be fundamentally connected, or aligned, to solve potential problems of overlaps, duplication of efforts and misalignment in the temporal scale of activities.

The benefits of this framework are threefold (Bigagli, 2017). First, the desired ‘link’ or synergy between the two sets of practices would reduce the current fragmentation of management. Second, AM's characterisation of ecological resilience would be improved through an improved coverage of socio-economic components. Third, TM's ecological considerations will be enhanced by the systematic inclusion of AM managers into transition arenas.

### 2.2 Case-study methodology

Specific research questions for investigation were formulated, related to each of the findings of previous research (Bigagli, 2015; see second column of Table 1). To answer to these questions, semi-structured interviews were conducted in the period June 2013–January 2014 with representatives of Italian public administrations and other subjects at various levels, engaged in the management of the marine environment or maritime activities of the Adriatic Sea. A total of 19 interviews were conducted, with 23 people: representatives of public administration, both at national and regional government level (16 people); representatives of scientific research bodies (4); relevant stakeholders in nature protection and fisheries (2); and international organisations involved in Adriatic cooperation (1). The full list of all subjects interviewed, together with a brief description and reasons for choice, is attached in Appendix. Because of their role or involvement, these people were expected to give the most complete picture about the current legal and policy practices and challenges for the management of the Adriatic Sea. The topics of discussion in the interviews were: their role within the institution; the competences and activities of their institution for the management of the marine environment and maritime activities; existing problems, challenges or good practices related to the governance of the marine environment and human activities, also in a coordinated way with other competent bodies; and existing problems, opportunities and challenges to the coordination of activities at Adriatic level. Transcripts were sent back to the interviewees shortly after the interview, to double-check the information included and reduce the risk of misunderstandings or lack of information. Information coming from the interviews was integrated with other documentary evidence, such as legal acts at national, regional and local level, other types of policy documents, like local plans and programmes of measures, and scientific research on the topic. The results of the case study investigation are presented in Section 4 and related sub-sections.

## 3 Results

### 3.1 The unit of management

1) Where is the MSFD implemented?

The MSFD identifies the Adriatic Sea as one of the marine sub-regions of the Mediterranean Sea marine region (art. 4). Accordingly, the Italian Ministry of Environment, responsible for the implementation of the MSFD, coordinated the elaboration of the initial assessment, of the monitoring programmes and of the Programmes of Measures (PoM), which considered the Adriatic Sea as one of the three Italian marine sub-regions where the MSFD is applied (the other two being the Italian sections of the Ionian Sea and of the Western Mediterranean Sea sub-regions). Italy's MSFD implementation covers the Italian jurisdictional waters of the Adriatic Sea, which include the territorial sea up to 12 nm from the coast and the continental platform, whose borders were agreed in several bilateral treaties with the then Yugoslavia (now valid for Slovenia and Croatia), Albania and Greece (Italy did not declare any Exclusive Economic Zone (EEZ) in the Adriatic Sea). In most cases, GES have been defined and environmental targets have been set for the whole of national marine waters, without distinction among marine sub-regions (Milieu, 2014).

2) Are there in place mechanisms for cross-border cooperation among countries sharing the same marine region or sub-region?

Interviewees involved into the implementation of the MSFD reported that coordination with other Member States (MS) is low or absent. Italy organized one meeting with Malta, Greece and Slovenia in 2012. These same interviewees pointed to the lack of a stable forum at basin-scale as a major obstacle for the coordinated implementation of the MSFD with other EU countries (Croatia, Greece and Slovenia). One interviewee highlighted that the EU institutions should assume a leading role in pushing Member States to cross-border cooperation for the implementation of MSFD.

3) Are there in place mechanisms for spatial coordination with other legislation?

**Fig. 2:**
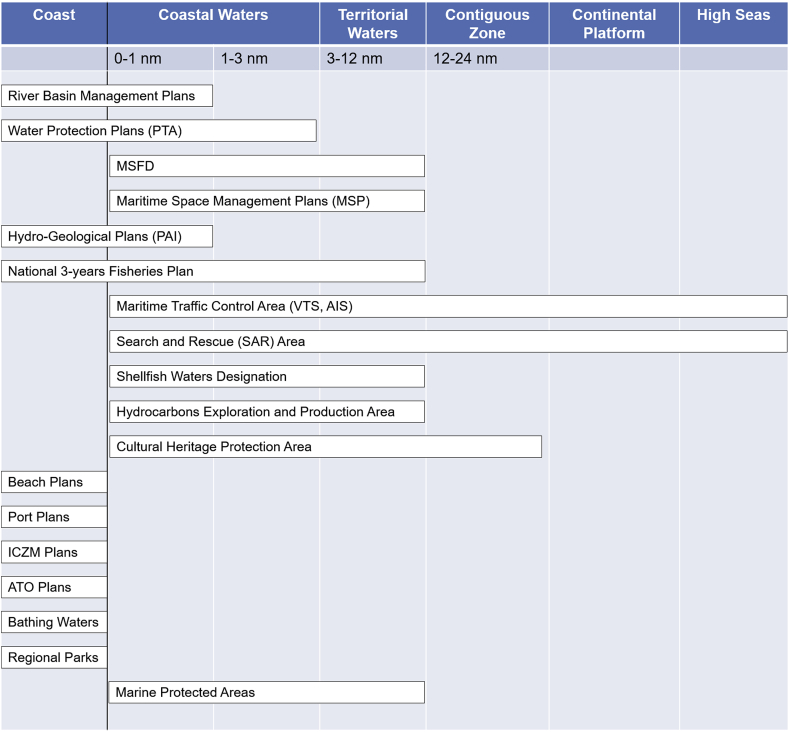
The spatial extension of the main plans and programmes in place in the Italian waters of the Adriatic Sea.

All interviewees reported the existence of several plans, programmes and policies in place for the Adriatic coastal and marine waters, each of them managed by a specific public body at various levels of governance. Their spatial scope is illustrated in Fig. 2, built on information coming from interviewees and from literature review; for an explanation of the acronyms used, see Table 2.

**Table 2:** Explanation of the acronyms used in Fig. 2, Fig. 3.

Acronym	
AIS	Automatic Identification System – automatic vessel tracking system
ATO	Optimal Territorial Area (*Ambito Territoriale Ottimale*) – Area of organization of integrated waste and wastewater public services; it is defined at regional level.
ICZM	Integrated Coastal Zone Management
PAI	Regional Hydro-geological Risk Plan (*Piano di Assetto Idrogeologico*)
PTA	Regional Water Protection Plan (*Piano di Tutela delle Acque*)
SAR	Search and Rescue
VMS	EU Vessel Monitoring System Regulation (2244/2003)
VTS	Vessel Traffic Service – marine traffic monitoring

Some of these plans, programmes and policies apply to coastal zones, as it is the case for e.g.: the regional Integrated Coastal Zone Management (ICZM) Plans; the ATO plans for wastewater management; the Port Regulatory Plans (for ports of national importance); and the coastal parks management plans. Others cover coastal waters, like the WFD River Basin Management Plans (RBMPs) and the Hydro-Geological Risk Plans (PAI), which extend to 1 nm offshore. Regional Water Protection Plans (PTA) extend the application of the WFD and other related Directives up to 3 nm offshore. Other plans, programmes and policies extend further into territorial waters; they are usually developed at national level and are under the responsibility of the competent Ministry. For example, the Ministry of Economic Development is responsible for the issuance of permits for prospection, exploration and production of oil and natural gas, in an area that extends up to the continental platform. Following the transposition of the MSP Directive, the Ministry of Infrastructures and Transport is required to implement Maritime Space Management Plans (MSMPs), which must cover marine waters (as defined by the MSFD), while excluding coastal waters that are already covered by other urban and rural plans. The Ministry of Agriculture and Forestry is responsible for the implementation of the Common Fisheries Policy (CFP), applicable to territorial seas and to fishing vessels flying the flag of a Member State. Maritime traffic control and Search and Rescue (SAR) operations are under the responsibility of the Coast Guard, and apply to the Italian SAR Area, which extends in the Adriatic high seas, up to the outer limit of the continental platform. Finally, it is worth noting that Italy established a contiguous zone, up to 24 nm from the coastline, for the protection of the marine environment from pollution and for protection and valorisation of cultural heritage.

Interviewees reported that all these policies are implemented in an autonomous way, where public bodies have generally no mandate to coordinate with others in relation to spatial measures. As such, each maritime activity is regulated independently, with provisions spanning across several European marine regions (as it is the case for the fisheries and maritime transportation sectors regulated by the Common Fisheries Policy, CFP), or at different governance scales. Several interviewees reported the absence of mechanisms for spatial coordination between these policies, with no coordination activities performed in the context of the implementation of the MSFD.

### 3.2 The objectives of management

1) How are GES identified and intended to be achieved?

GES and environmental targets have been defined by the Italian Ministry of Environment in the initial assessment (ISPRA, 2012), with the scientific, technical and coordination support of the Italian National Institute for Environmental Protection and Research (ISPRA). They have been refined with the publication of monitoring programmes in 2015, after a public consultation held in June 2014 (available online at: http://www.strategiamarina.isprambiente.it/sintesi-risultati-consultazione-2014), to which a total of 677 respondents participated. Italy reported 7 monitoring programmes, covering only the MSFD elements, criteria and indicators that have been evaluated as important and appropriate for the characteristics of Italian marine waters and in relation to the human activities and resulting pressures. The recent evaluation of MSFD monitoring programmes by the European Commission (European Commission, 2017) acknowledges that most Italian monitoring programmes are consistent with previously identified GES and targets, with some improvements needed for specific descriptors (namely Descriptors, 2, 3, 5, 8, 9 and 11). Moreover, limited information was reported on the monitoring of transboundary impacts (European Commission, 2017).

The MSFD PoM has been published in 2016, after a public consultation held in October 2016 (available online at: http://www.strategiamarina.isprambiente.it/ConsultazionePoMott2016_Esito.pdf), to which 5 respondents provided comments. The PoM contains a list of existing measures, currently adopted in the framework of other international, EU and national legislation, as well as a list of new measures to implement, in order to fill the gaps left by the existing ones. A list of all existing and new measures included in the PoM is given in Table 3.

**Table 3:** Links between existing measures included in other policies and MSFD Descriptors.

MSFD Descriptors	List of existing measures	New measures
Descriptor 1 (Biodiversity) Descriptor 4 (Food Webs)	Natura 2000 implementation measures (Habitats and Birds Directives); reduction of by-catch of cetaceans (Regulation 812/2004); National Biodiversity Strategy	Measure 1 – Extension of Natura 2000 network to marine areas Measure 2 – Solutions to reduce collision with cetaceans Measures 4, 5 and 6 – Measures to reduce by-catch of elasmobranches, sea cetaceans and sea turtles, and marine birds Measure 7 – Measures against damage to benthic habitats and species
Descriptor 6 (Seafloor Integrity and Habitats)	National Energy Plan; safety and environmental impact of hydrocarbon activities (Directive 2013/30); national law on port dredging and disposal (Decree 152/2006); EIA and SEA of related activities and policies; CFP	Measure 7 – Measures against damage to benthic habitats and species Measure 9 – Guidelines on sealing over biogenic layers
Descriptor 2 (Invasive Species)	Regulation on alien species in aquaculture (708/2007); National Strategic Aquaculture Plan; Regulation on invasive alien species (1143/2014)	Measure 8 – Creation of a National Focal Point on Harmful Aquatic Species and Non-Indigenous Species
Descriptor 3 (Commercial Fish Stocks)	CFP and National Fisheries Plans	
Descriptor 5 (Eutrophication)	Regional PTAs (WFD); national laws on effluents from livestock (Decree 7/2006) and on impacts from aquaculture (Decree 508/2014)	
Descriptor 7 (Hydrographic Conditions)	WFD; EIA and SEA of related activities and policies; Regional ICZM Plans; MSP	
Descriptor 8 (Contaminants)	WFD; REACH; Biocides Regulation (508/2012); Regional PTAs (WFD)	
Descriptor 9 (Contaminants in Seafood)	EU Regulations on food hygiene and health	
Descriptor 10 (Marine Litter)	EU Directives on waste from ships (2008/98 and 2000/59); Regional Waste Management Plans (not mentioning marine litter)	Measures 10 and 11 – Measures to reduce marine litter from fishing activities Measure 12 – Education and literacy on marine litter
Descriptor 11 (Energy and Underwater Noise)	EIA and SEA of related activities and policies	

The lack of the right quantity and type of data and information was indicated by one interviewee as one of the main reason for the adoption by Italy of qualitative criteria to identify GES and environmental targets. The same interviewee pointed to the ambiguity and vagueness of MSFD legal requirements on this aspect as a further obstacle.

The socio-economic analysis of the use of marine waters, required by the MSFD, was performed by Italy using the Marine Water Accounts approach, which included three types of macro-economic data for each maritime sector: production value, added value and employment. Moreover, the analysis of the cost of degradation of the marine environment was performed using the Cost-based approach, where the expenses of various public bodies for remedying environmental damage and impacts are included. While, as acknowledged in the Italian initial assessment, this provides a good snapshot of actual costs sustained to remedy environmental damage to the marine environment in Italy, however Italy acknowledges that it misses the loss of social wealth derived by the degradation (ISPRA, 2012).

2) Have objectives been set at Adriatic level, or at the scale of other related SESs?

The only experience of coordination of management at institutional level was reported by one interviewee, and is related to the Fisheries District of Northern Adriatic. Launched in 2010 and operative from 2012, it aims at coordinating the management of fish resources of the Northern Adriatic Sea, by promoting partnerships between producers and enterprises of the fisheries sector. The spatial scope of the District includes the marine waters of the three northernmost Italian Adriatic regions: Friuli-Venezia Giulia, Veneto and Emilia-Romagna, as well as Croatian and Slovenian marine waters. Cooperation was reported also at project-level, with the two EU-funded projects AdriPlan and DeFishGear. The aim of AdriPlan (2013–2015) was to analyse and promote Maritime Spatial Planning in the Adriatic-Ionian region; it developed proposals and recommendations for an operational cross-border MSP process, ensuring among others the good status of marine ecosystems and the provisioning of ecosystem services (Barbanti et al., 2015). In this line, the national law of transposition of the MSP Directive envisages cross-border coherence and coordination through existing national competent authorities and trans-national initiatives. The aim of DeFishGear (2013–2016) was to facilitate efforts for integrated planning to reduce the environmental impacts of litter-generating activities and ensure the sustainable management of the marine and coastal environment of the Adriatic and Ionian Seas (see the project website: www.defishgear.net). Another experience of coordination at the scale of marine region is the UNEP/MAP-led EcAp project, for an Ecosystem Approach to the Mediterranean Sea, based on the provisions of the MSFD and, marginally, of the WFD. Some interviewees acknowledge the importance of this initiative in the establishment of a framework for discussion on these issues, especially in combating episodes of pollution discharge, reported to take place in high seas and in other countries, where legislation and control of competent authorities is less stringent. However, the same interviewees also underline that major threats to EcAp implementation lay in the varying level of commitment to the project by the single countries, especially outside the EU, and in the lack of effective mechanisms for enforcing compliance. One interviewee argued that the presence of different legal systems among Mediterranean countries for ship-generated waste management, often results in ‘dumping’ practices, where ships discharge their waste in a faster, easier and cheaper way in countries with lower environmental standards, with increased risks of damaging the marine environment. Common rules are envisaged for the fisheries sector by another interviewee, especially in relation to the protection of nursery sites and to a clearer delimitation of fishing grounds.

3) Have the objectives of other legislation been integrated with MSFD into a common framework?

**Fig. 3:**
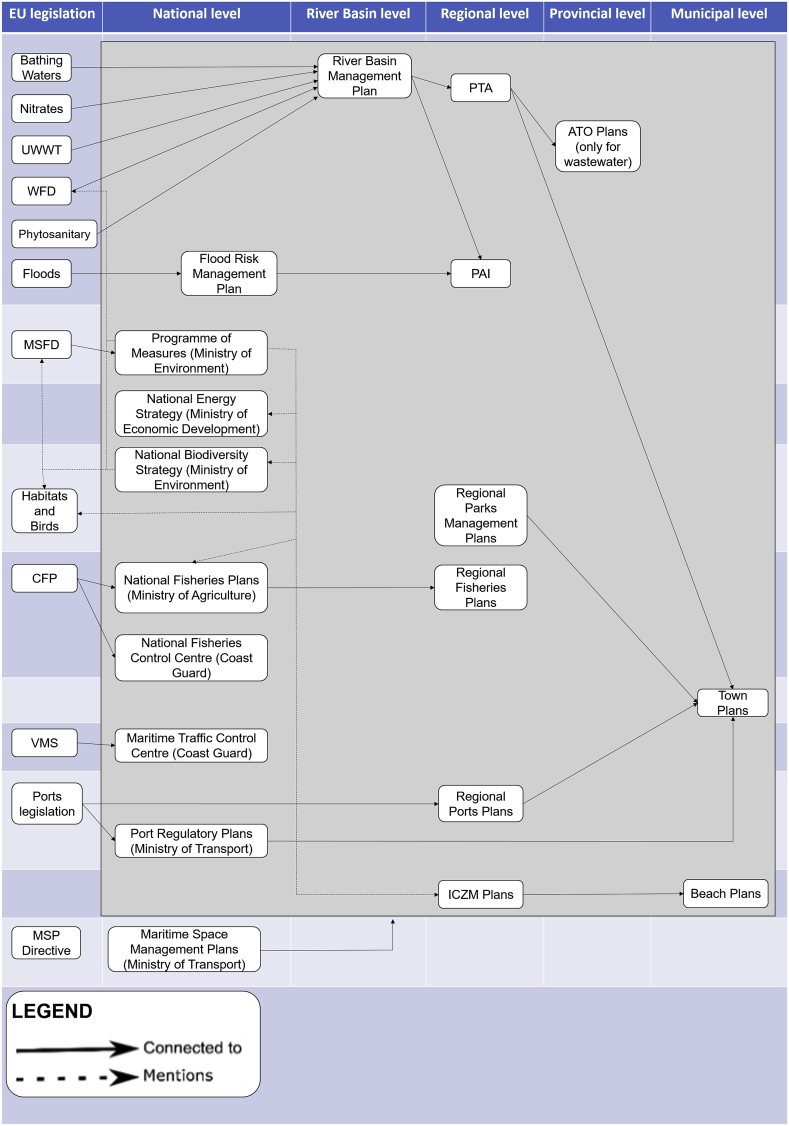
Connections between EU legislation and existing policies, plans or programmes for the Adriatic Sea.

All interviewees reported the existence of several plans, programmes and policies, implemented by several bodies in an autonomous way and with no mechanisms in place for the coordination of objectives. Some plans try to coordinate the implementation of multiple EU legal acts, the most notable example being the regional PTAs aiming at the good ecological and chemical status of surface waters (following the provisions of the WFD and related legislation). Coordination is in place also for the regulation of port activities, where the Port Regulation Plan should be developed in synergy with local town plans. Coordination is envisaged also by the Decree 201/2016 of transposition of the MSP Directive. Art. 5.3 of the cited Decree requires existing plans and programmes on marine waters and related economic and social activities, as well as the ones that are relevant for land-sea interactions, to be included and harmonised with the Maritime Space Management Plans (MSMPs). The MSMPs have not been elaborated yet at the moment of writing. The general formulation included in the text of the national Decree allows for the potential inclusion and harmonisation with any of the existing plans and programmes, related to the activities listed in art. 5.1 (aquaculture and fisheries; oil and gas prospection, exploration and exploitation and renewable energy production; maritime transport routes and traffic flows; military training areas; nature conservation and protection areas; raw material extraction zones; scientific research; underwater cables and pipelines; tourism; and underwater cultural heritage). Another example is the National Biodiversity Strategy, elaborated and implemented by the Ministry of Environment in 2010, which aims at creating a national platform for the protection of biodiversity across socio-economic sectors, including marine biodiversity. The Strategy identifies objectives and actions to be implemented, linked to WFD, MSFD, CFP, energy and tourism legislation, in order to integrate the protection of biodiversity into sector policies. It is worth noting that the Strategy does not create new obligations; it only asks to consider the protection of biodiversity while implementing other policies. Finally, some regions have adopted Integrated Coastal Zone Management (ICZM) Plans, aiming at coordinating the management of multiple activities and interests in the coastal zone. However, as some interviewees highlighted, some of them, like the Marche region ICZM Plan, focus on specific issues like erosion and coastal defence, while others, even if with a larger scope, are just a set of non-compulsory guidelines, for which one interviewee pointed to the need for more cogent legislation (like in the case of the Emilia-Romagna region ICZM Plan). Existing plans, programmes and policies for the marine environment and maritime sectors, including their relations, are illustrated in Fig. 3; acronyms are explained in Table 2.

The MSFD PoM tries to integrate the objectives of other policies for the purposes of the achievement of GES. It does so by including a list of existing measures, taken in the context of other international, EU and national policies, which can contribute to the achievement of GES for Italian marine waters. This list is integrated by a set of 11 new measures, aimed at filling the gaps identified through a gap analysis. The detailed links between existing and new measures with MSFD Descriptors are illustrated in Table 3. The public consultation on PoM included suggestions of improvement of new measures. One respondent highlighted the importance to include measures of prevention, rather of management, of invasive species, pointing to the need for Italy to ratify the 2004 International for the Control and Management of Ships' Ballast Waters and Sediments, which is in force since September 2017.

**Fig. 4:**
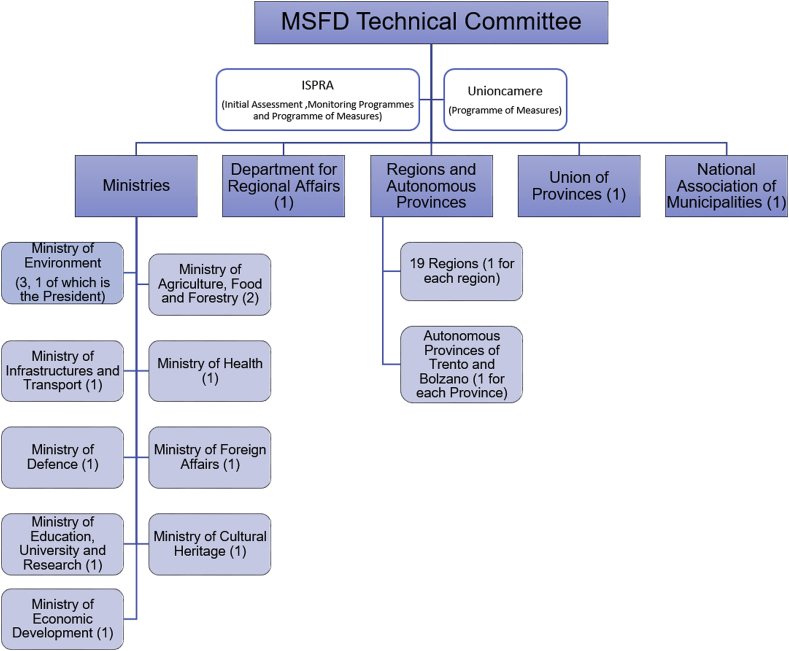
The composition of the MSFD Technical Committee (in parenthesis, the number of representatives).

In a parallel way, at institutional level, a major effort in trying to build a platform for cross-sector management of the marine environment is the Technical Committee for the coordinated implementation of the MSFD. The Technical Committee was established in 2010; it is composed of representatives of Ministries, Regions, Provinces and Municipalities (see Fig. 4). Its aim is to participate to the definition of all the activities of implementation of the MSFD. A further platform for cooperation was piloted in 2012 with a Protocol of Understanding between the Ministry of Environment and the Italian coastal regions, grouped by marine sub-region, and aiming at integrating the initial assessment with investigation on three priority themes (marine litter, habitats and species and socio-economic aspects). A Coordination Body (Cabina di regia) was created for the Adriatic sub-region for the period 2012–2013, which reportedly represented the first attempt to establish a platform for cooperation and coordination of activities among public bodies for the marine environment. However, interviewees reported a low level of interest and involvement on the activities of the Technical Committee from non-coastal regions. One interviewee pointed out that the process of implementation of the MSFD needs time and resources, and that all public administrations involved at all levels need to learn to collaborate, with the objective of building a stable structure and a common methodology of work. In this respect, the public consultation on MSFD Monitoring Programmes highlighted the need to foster the inclusion of environmental associations, tourism and fisheries operators, as well as marine sports associations into the institutional mechanisms of governance for the MSFD.

Low or absent coordination was reported by the interviewees, not only between different levels of authority (state; regions; provinces and municipalities), but also inside the same public body. For example, departments of the Regions that are responsible for erosion, coastal defence and ICZM, are usually not involved in the activities of the MSFD, and vice versa. One interviewee reported that current legislation does not include provisions for the coordination of dredging activities among the responsible bodies, i.e. Regions and National Port Authorities, especially in relation to Sites of National Importance (particularly polluted areas identified at national level). Moreover, regional River Basin Authorities, which are responsible for the implementation of flood risk management activities, have no coordination with tourism or coastal public area managers, let alone the two departments mentioned above. Cooperation among regions is reported by interviewees as left to personal relations between the single actors. It is the opinion of one interviewee that coordination among public bodies is hampered by the quantity of laws and regulations, which often generates confusion instead of clarifying competencies. The institutional setting of the RBDs adds to this complexity and fragmentation. A positive sign in this direction, after several years of delay, is the establishment by the Ministerial Decree no. 294 of 25 October 2016 of the three missing River Basin Authorities (Northern Apennines; Central Apennines; and Southern Apennines), following the provisions of the WFD. Consequently, the River Basin Authorities of rivers Arno and Tevere (two rivers flowing into the Tyrrhenian Sea), originally required by law to temporarily perform the duties to be assigned to the Northern and Central Apennines RBDs, were eliminated. The cited Ministerial Decree came into force in February 2017, and its implementation is on its early stage. Much has yet to be seen on the relationships between the governance configuration of river basins on the one side, and the one created for the implementation of the MSFD on the other side, especially in relation to overlaps and potential conflicts of objectives between the two Directives.

### 3.3 The structure of management

1) How is the MSFD policy cycle implemented?

Italy reported the initial assessment and definition of GES and environmental targets to the European Commission in 2012 (ISPRA, 2012), with an integration of missing data and information dated April 2013. Next, monitoring programmes were elaborated and reported in 2015, while the Programme of Measures was reported in 2016. Both the initial assessment and the elaboration of monitoring programmes have been elaborated starting from the knowledge available and accessible by the Ministry, building on existing data held by, and existing monitoring programmes conducted by, other Ministries or other public bodies, such as regions and national research bodies, and with the technical support of the National Environmental Agency (ISPRA). The Monitoring programmes and the Programme of Measures were developed by the Ministry of Environment and approved by the Technical Committee, with the technical support of Unioncamere (the Union of Italian Chambers of Commerce) for the PoM. In the case of monitoring programmes, the intention of the Ministry of Environment was to integrate existing data collection activities with new monitoring, aiming at filling the data gaps identified in the initial assessment. Italy's monitoring programmes are reported to be operational by 2018, due to lack of allocation of responsibilities and financial commitment (Milieu, 2014), in contrast with the deadline set by MSFD art. 5(2) of 15 July 2014 (as noted also by European Commission, 2017). Moreover, while Italy identified some monitoring gaps for specific descriptors (3, 5, 8, 10 and 11) and proposed a plan to address them, however plans to address such gaps for Descriptors 1, 2, 4 and 6 are still missing (European Commission, 2017). To this respect, two interviewees highlighted the need for further coordination on data collection and monitoring activities among deputed bodies, especially in relation to the evaluation of the status of habitats and species for the requirements of Habitats and Bird Directives. Another interviewee reported a structural lack of data in the hands of Regions, which hampers the development of adequate knowledge in support to MSFD implementation.

The major obstacles being faced in the implementation of the MSFD, as reported by interviewees, are: the lack of funds and staff; the lack of precise addresses and strategies at national level; the differences in legal frameworks among the Italian Adriatic regions, which may slow down the activities or require their adaptation to different sets of rules; the overlaps and misalignments of various legal acts, especially at EU level; and the fragmentation of duties and responsibilities, not only among different bodies, but also among different departments or services of the same body (e.g. Ministry, Region).

2) Are there initiatives and mechanisms in place to foster the coordination of MSFD with other legal acts?

Overlaps in competences and relative isolation of policies have been highlighted by interviewees, with no connection with, and sometimes reported knowledge of, the implementation of the MSFD (one interviewee). Interviewees reported the existence of different calendars and deadlines for the implementation of each policy, plan or programme, creating sometimes difficulties in the organisation of the workload and the capacity of the public body to fulfil the requirements. This is especially true for these regions, whose territory lays within the area of multiple RBDs, and where RBMPs are formulated and implemented in an autonomous way, without coordination or cooperation with other marine plans and programmes. The governance is particularly complex in the case of some sectors, like the wastewater management. As reported by one interviewee, several bodies are involved at various levels (regional; provincial; supra-municipal and municipal levels) in wastewater management, and the capacity of the regions to coordinate them was reported as low.

For the purposes of implementation of the MSP Directive, two institutional mechanisms will be established. The first is an Inter-Ministry Coordination Table on MSP, composed of representatives of several Ministries, including the Ministry of Environment, of the Department for Regional Affairs and of the Customs Agency. It defines the guidelines for the implementation of MSMPs in coordination with other existing plans. The second is a Technical Committee, with the objective of drafting MSMPs, composed of representatives of a restricted number of Ministries (among which the Ministry of Environment). When established, these two new institutional mechanisms will have the potential to contribute to the integration of the MSFD objectives into other existing maritime policies, plans and programmes.

## 4 Discussion

### 4.1 The limitations of the MSFD and suggestions to strengthen its implementation

The analysis of the legal and policy landscape for the governance of the Adriatic Sea, presented in this paper, showed the importance of the MSFD as the first policy trying to deliver an integrated, complex systems approach to the assessment and management of European oceans and seas. For the first time in Italy an assessment of the status of the marine environment was performed, based on the operationalisation of the concept of ecological resilience set up by the MSFD through the GES criteria, descriptors and indicators. This assessment had a geographical, social-ecological approach as it targeted the marine sub-regions identified through the MSFD. Finally, Italy built institutional mechanisms and structures for the coordinated implementation of the MSFD. However, the present case study investigation confirms the three limitations of the MSFD, presented in Section 1 (Bigagli, 2015).

First, the MSFD seems to provide an insufficient geographical approach to EU marine regions and sub-regions. Italy implemented the MSFD in an autonomous way, without much coordination at Adriatic level, as reported by several interviewees. Moreover, Italy's MSFD Monitoring programmes did not give enough coverage of transboundary impacts, as noted by the European Commission (2017). Overall, this stands as another case of ‘nationalisation’ of the implementation of the MSFD, as stated by Freire-Gibb et al. (2014). Moreover, the implementation of the MSFD in Italy for the Adriatic Sea seems to lack the creative thinking on future options for cross-border cooperation, which emerges from the analysis of other marine sub-regions of the Mediterranean (Jouanneau and Raakjaer, 2014). This seems to be mainly due by the fact that cross-border, marine region-based cooperation is formulated in a general way in the text of the MSFD, without much detail on governing structures and mechanisms (as found also by Salomon and Dross, 2013, Van Tatenhove et al., 2014, Van Tatenhove, 2016). This is in line with findings of Cavallo et al. (2016), who attribute the low level of cross-border coherence and coordination to the MSFD being a ‘Framework’ Directive (see also Boyes et al., 2016). As a result, MS are left to the capacity of the Regional Sea Conventions (RSCs) to act. It is true that the EcAp project of the UNEP/MAP is one of the most advanced examples of the application of a complex systems approach to marine assessment and management (Bigagli, 2016). However, the present research confirms the findings of Van Leeuwen et al. (2012), who stated that the institutional ambiguity of the Mediterranean governance framework is the highest in Europe, as support from the RSC is lacking. The findings presented in this article point to two main limitations of the EcAp approach, mainly lying in the low interest of non-EU Mediterranean countries to support its implementation, and the lack of mechanisms to enforce compliance.

Second, the vagueness of the MSFD is confirmed as an obstacle to the process of performing the required socio-economic assessment, and it's linking to the GES assessment. The MSFD requirements related to the socio-economic analysis were evaluated by the interviewees as general and vague; this led Italy to adopt the methodology that best suited data availability. The result is that important aspects, such as the complex dynamics between socio-economic activities and their impacts on marine ecological resilience, or the assessment of marine ecosystem services and benefits, or cross-scale interactions and effects, were not adequately taken into consideration, not only in the socio-economic assessment, but also in the assessment of GES and setting of environmental targets, as well as in the elaboration of the Monitoring programmes and the Programmes of Measures. This confirms findings from other research, on the lack of clarity of the meaning of GES (Brennan et al., 2014), and on its low level of accuracy and strategy at technical level (De Jonge et al., 2012, Bellas, 2014). Another aspect that emerges from the present analysis is that Italy performed a qualitative assessment of GES, mainly because of the lack of the right quantity and quality of data. As pointed out by various interviewees, and acknowledged also in literature (Tunesi et al., 2013), this structural lack of data is not only related to the insufficient, or inexistent monitoring of several ecological structures and processes, but also to an insufficient level of sharing of data among public administrations. Italy adopted important initiatives to fill the data gaps, by establishing the cited Cabina di Regia in 2012 and by identifying plans to address monitoring gaps for certain specific Descriptors (European Commission, 2017). However, further efforts on data sharing should be carried out, especially in relation to the implementation of the INSPIRE Directive (Directive 2007/2/EC) establishing an infrastructure for spatial information in Europe, in the marine domain. As showed by Abramic et al. (2015), the implementation of INSPIRE has the potential to ‘untap’ existing data held by public administrations at various levels, removing obstacles to their availability, share and reuse, and thereby benefiting Member States in their implementation of environmental policies.

Third, the MSFD seems to suffer from an insufficient capacity to coordinate with other laws, policies and programmes in place at various levels. The case study of the Adriatic Sea showed a high level of fragmentation of marine governance, with several public bodies responsible for specific areas, laws or policies at various levels (EU, national, regional and local), with little or no collaboration and cooperation among regions, as highlighted also by Hummel et al. (2015). Moreover, the implementation of the EU legal acts is negatively affected by the presence of different time calendars and deadlines to achieve the various environmental targets, as highlighted also by Boyes et al. (2016). To this respect, it is worth noting how Italy reported the implementation of the MSFD Monitoring programmes by 2018, in contrast with the MSFD requirements for monitoring programmes to be implemented by 2014.

Thanks to the process of implementation of the MSFD, some initiatives are slowly starting to take place. At institutional level, the creation of the MSFD Technical Committee goes in the direction of involving different authorities at various levels of governance into the implementation of the MSFD. Moreover, the MSFD PoM identified existing measures, being taken in the context of other policies, which would contribute to the achievement of GES, as well as new measures to fill the gaps identified. However, some issues should be highlighted. First, reference by the PoM to other legislation is often too descriptive and general. Second, as highlighted by one respondent to the public consultation on Italy's monitoring programmes, the implementation of the MSFD would benefit from an extended involvement and participation of stakeholders, like marine environmental protection associations, tourism and fisheries operators and marine sports associations. Third, the issue is open also in Italy on whether the objectives of GES will be given priority in case of conflicts with other socio-economic objectives (e.g. Blue Growth Agenda) (Boyes et al., 2016). It is true that the MSFD Technical Committee provides a forum for the presentation and possible solution of such conflicts. However, by including sector-based policies into the implementation of the MSFD, and not vice versa, as suggested also by Salomon and Dross (2013), the risk is that MSFD GES objectives will not be part of the policy formulation and implementation of the relevant, and relatively isolated, maritime sectors. An opportunity to solve this issue may come from the implementation of the MSP Directive. Both the Inter-Ministry Coordination Table and the MSP Technical Committee have the potential to provide a more coordinated management of all marine and maritime policies, and to integrate the GES into sector-based policies. However, the question is open on whether the whole MSP process will simply add another parallel mechanism alongside the existing ones, thereby incrementing the complexity of the governance and adding further different deadlines of actions, which would overlap with existing ones.

In the light of these findings, what can be done to strengthen the implementation of the MSFD?

First, there is the need to foster mechanisms and structures for cross-border coordination at the level of marine regions and sub-regions. Adriatic cooperation could be improved by setting up a permanent table of coordination at marine sub-region level, involving other Adriatic EU and, possibly, non-EU countries in the implementation of the EcAp project. In this context, the EUSAIR can play a primary role, by directing funding on marine environmental cooperation in support of Adriatic countries. Second, existing efforts on improving marine data access, sharing and re-use should be fostered, integrating also socio-economic data. A more coordinated implementation of existing data policies and initiatives, like INSPIRE and EMODnet (Millard et al., 2015), as well as the building of a Mediterranean marine SDI (Cinnirella et al., 2012), would contribute to an improved quality of data and information, underlying all phases of the implementation of the MSFD. This should be accompanied by efforts to foster scientific research to fill data and knowledge gaps, especially for ecological structure and processes that are under-monitored, like marine food webs, litter and underwater noise. Possible solutions could come from cross-border integration of monitoring, as indicated also by Zampoukas et al. (2013), or by combining different tools to strengthen marine data collection practices, like genomic tools, remote sensing, acoustic devices and modelling (Borja et al., 2016a). Third, solutions to improve coordination among sectors and organisational levels should be promoted, for example through the integration of the objectives of the MSFD into sector policies, or the introduction of a ‘marine ecological resilience assessment’ for every policy or activity that is likely to have an impact on the marine environment. Moreover, inter-sector coordination units could be set among departments and units of the same public administration (e.g. a same Ministry or Region), possibly involving other bodies at lower levels (e.g. provinces and coastal municipalities), in a way similar to the Technical Committee already in place in Italy, in order to foster collaboration and cooperation. Further opportunities could come from the implementation of the Maritime Spatial Planning Directive (2014/89/EU), where such plans could act as a forum for participatory discussion and building of a shared vision of sustainability for a marine sub-region or area.

### 4.2 Is it possible to implement a complex adaptive systems approach to marine systems?

Three main challenges emerge from the findings of the present research, related to the capacity of the framework proposed to be applied into legal frameworks and management practices of marine complex adaptive systems. They are summarised in the first column of Table 4, and briefly discussed below.

**Table 4:** Challenges in implementing the proposed framework and suggestions to improve legislation and management practices, for each component of the framework proposed.

	**Theory** Obstacles in implementing a complex systems approach to marine systems	**Management Practices** Possible actions to remove the obstacles identified in theory
Unit of management	It is difficult to set up clear boundaries to the systems to assess and manage, as socio-technical systems are not place bound	Soft-law or project-based initiatives to extend assessment and management beyond jurisdictional waters Foster international cooperation through formal or informal institutional mechanisms at marine-region level
Objectives of management	The assessment of ecological resilience may be highly challenging and impossible to achieve	Support the ecological resilience assessment through improved guidance at EU level and through the use of existing integrative tools (e.g. CHI, OHI) Improve marine and maritime data collection and monitoring, access and sharing
Structure of management	Relative isolation of AM and TM approaches, with different visions on the management of conflicts among principles and objectives	Introduce a “marine ecological resilience impact assessment” Support the establishment of formal or informal institutional mechanisms for cross-sector management (including provisions to align conflicting deadlines)

A first challenge in implementing this approach is related to the level of unit of management. As socio-technical systems are not place-bound but span across multiple social-ecological systems, without setting clear boundaries to the system to analyse and manage (be it a social-ecological or a socio-technical system), too many processes and interactions should be covered, with considerable effort required and questionable added value for effective management. What emerges from the analysis of legislation (Bigagli, 2015) and the present case-study investigation is that TM's conceptualisation of socio-technical systems, actors and institutions may be a useful heuristic tool to support managers into the development of policy options and management measures, but may not be easily used, as practical computational applications built on this have not been developed yet, as acknowledged by Van der Brugge and Van Raak (2007). A possible solution to this issue could be to clearly define the scale of action, as suggested also by De Jonge et al. (2012), establishing clear links, mechanisms and procedures for continuous exchange of knowledge between the two sets of managers during all phases of systems assessment.

The second challenge relates to the objectives of management, namely to the notion of ecological resilience and the capacity to assess it. Several voices in scientific literature highlight the difficulties in assessing ecological resilience. Eason et al. (2016) point to the difficulty to identify critical variables driving system transitions and shift towards different status, such that there may never be the capacity to fully quantify the ‘resilience’ of a social-ecological system. There is currently no consensus among ecologists on basic concepts and on related assessment indicators. As highlighted by Borja et al. (2016b), there are currently no methods to assess marine health in a holistic way, integrating information from multiple ecosystem components, nor methods to evaluate cumulative effects of multiple pressures. Along the same line, De Jonge et al. (2012) highlight the fact that ecologists do not agree on a common integrated concept of ecological complexity, which adds to the pressure from decision makers for environmental indicators that are ‘easy to understand’ and ‘cheap and simple to measure’. The framework gives for granted the possibility to have a full understanding of marine ecological resilience, but, in line with cited literature, both previous research (Bigagli, 2015, Bigagli, 2016) and the results of the present research seem to confirm this difficulty. The notion of GES of the MSFD lacks the inclusion of important components of social-ecological systems, like ecosystem services and human benefits (Bigagli, 2015). The analysis of the case study of the Adriatic Sea showed that the vagueness in the text of the MSFD, as well as the lack of guidance from the EU institutions led Italy to adopt a qualitative approach in the identification of GES and environmental targets, while other MS have used different methodologies (Palialexis et al., 2014). Moreover, Italy's monitoring programmes were evaluated as too general from both Palialexis et al. (2014) and Hummel et al. (2015), who confirm that the real weakness is the lack of operational indicators for MSFD monitoring. In addition to this, Italy used the Marine Waters Account and the Cost-Based Approach methodologies for the socio-economic assessment required by the MSFD. This confirms the limitations attributed to literature, on the lack of attention of AM to not only micro-level, but also to several macro-level socio-economic components. As reported by Borja et al. (2016a), there is an ongoing debate at European level on the opportunity to have a ‘pass/fail’ approach, or to assess each GES Descriptor independently. In this line, potential contributions towards the solution of this issue come from the work of De Jonge et al. (2012) and Pinto et al. (2013), whose integrative assessment framework, combining DPSIR with the Ecosystem Services approach and Ecological Network Analysis (ENA), shows the capacity to effectively integrate ecological and socio-economic data and information, needed to implement the desired complex adaptive system (i.e. EBM) approaches in an effective way.

The last challenge lies at the level of structure of management. Relatively isolated policies and institutional structures and mechanisms for their implementation in the Adriatic, reflect the relative isolation on the conception and development of both AM and TM, who currently pay little or no attention to the issue of integrating management across sectors, themes and issues. The MSFD has a soft stand on this issue, asking to ensure that the “collective pressure of maritime activities is kept within levels compatible with the achievement of good environmental status and that the capacity of marine ecosystems to respond to human-induced changes is not compromised, while enabling the sustainable use of marine goods and services by present and future generations” (art. 1(3)). And practice shows how public administrations are organised following a “Weberian” model where complex problems are split into smaller, more digestible bits, each of them being assigned to a different administration or working group inside a same public body. Some initiatives try to fill this gap and reduce fragmentation, like the MSFD Technical Committee in Italy. However, AM would expect MSFD managers to participate to sector-based policy formulation and implementation, and not vice versa, as found in practice. The result is that the MSFD remains as another sector-based policy, struggling to build bridges across sectors and levels of governance.

More generally, both AM and TM seem not to pay enough attention to the possibility of conflicts between the objectives of management across scales. Given that the two theoretical approaches have a different vision of sustainability, which vision should be prioritised? What if, for example, the transition of the EU energy sector to a low- or no-carbon model would include the promotion of offshore wind farms, with negative impacts on marine habitats and species (Fox et al., 2006, Wing Goodale and Milman, 2014) and on the achievement of MSFD environmental targets? In a similar way, how bearable are the effects of Carbon Capture and Storage (CCS) activities, developed in order to mitigate the impact of anthropogenic emissions of greenhouse gases into the atmosphere, to marine biogeochemical cycles and biodiversity (Blackford et al., 2009)? Neither AM and TM, nor the framework developed seem to shed light on this respect. A possible solution could come from the systematic inclusion of AM (i.e. MSFD) managers into the arenas of specific sectors (e.g. fisheries, maritime transportation) in order to foster the respect of ecological resilience and environmental boundaries. The clear advantage would be that sector-based management would acquire a clear context and a clear direction, i.e. towards the respect of ecological boundaries. However, seen from the perspective of TM, this could be seen as a tentative to “capture” the arena by powerful incumbents of the status quo, a vulnerability already acknowledged in literature especially for the Dutch energy sector (Kern and Smith, 2008, Smith and Kern, 2009, Voss and Bornemann, 2011).

## 5 Conclusions

The failure of traditional, sector-based management calls for the implementation of complex adaptive systems approaches to the assessment and management of marine systems. This paper presents the results of a case study research, aimed at investigating whether the limitations of related EU legislation are translated or not into the practice of the case study area of the Adriatic Sea, with the purpose of deriving lessons on the real possibilities to implement a complex adaptive systems approach to the assessment and management of marine systems.

Results show that the MSFD triggered important efforts at all levels of governance, introducing a geographical, learning-based approach for the achievement of the GES of EU marine systems. However, this research confirmed the three major challenges, related to the unit, the objectives and the structure of management, and suggests possible strategies to tackle these challenges for the Adriatic Sea.

These difficulties highlighted for practice pair up with the identification of three major obstacles and weaknesses of complex adaptive system theoretical approaches, which need further attention and development, especially in relation to marine systems.

## Appendix

### List of organisations interviewed

**Adriatic-Ionian Initiative (AII)**

*No. of people interviewed: 1*

The Adriatic-Ionian Initiative (AII) is an initiative of foreign policy promoted by the Italian government, and targeted at building a permanent table of cooperation and peace with other countries of the area, promoting the Adriatic-Ionian Macro-Region. The Secretariat of the AII has the function of giving continuity to the political activities of AII countries, by providing administrative and documental support to the Ministries of Foreign Affairs. AII funds micro-projects at 50% in various themes, including on the environment (Regional Cooperation Programme).

The AII was chosen because of its activities in support to cross-border coordination on the marine environment and maritime sectors at Adriatic level.

**Ancona Port Authority**

*No. of people interviewed: 1*

The port of Ancona is one of the biggest Adriatic ports in terms of transit of maritime passengers on ship lines, excluding cruise ships passengers. The Ancona Port Authority (PA) is responsible for the following activities: planning, programming, coordination and control of port operations, and of the other commercial and industrial activities performed in port; supervision, management and regulation of land-based port facilities and services; maintenance of the common spaces in the port areas, including the depth of the port basins; appointment and control of the activities of port operators.

The PA was chosen because it is responsible for the implementation of existing legislation on maritime transport and environmental protection in the port.

**Harbourmaster Corps – Coast Guard**

*No. of people interviewed: 1*

The main duties of the Corpse are the safeguard of life at sea, the security and safety of maritime transport, the protection of the marine environment and marine ecosystems, and the surveillance over the fisheries sector.

The Corpse depends functionally from three Ministries: the Ministry of Infrastructures and Transport; the Ministry of Agriculture, Food and Forestry; and the Ministry of Environment, Territorial and Sea Protection.

The Coast Guard was chosen because of its responsibilities in the implementation of existing legislation regulating maritime transport, fisheries and marine environment protection.

**Marche River Basin Authority**

*No. of people interviewed: 1*

The Marche Region River Basin Authority is responsible for the implementation of regional policies in the fields of hydro-geological and floods risk management.

The River Basin Authority was chosen because it is involved in the assessment and management of coastal floods and river basin management, which includes coastal waters.

**Ministry of the Environment, Territory and Sea Protection**

*No. of people interviewed: 1*

The Ministry of the Environment is responsible for the implementation of environmental policy at national level.

The Division of Coastal and Marine Environment Protection was chosen because it is responsible for the implementation of the MSFD at national level.

**Mount Conero Regional Natural Park**

*No. of people interviewed: 2*

The Regional Park of Mount Conero is a coastal protected area in the territory of the Marche region, whose management is regulated at regional level. It covers a sea strip delimited by the foreshore line, at the depth of 6 meters. The Park includes three coastal Sites of Community Importance (SCI), designated in the framework of the Natura 2000 network.

The Conero Park was chosen because it is a protected area on the coasts of the Adriatic Sea, contributing to the implementation of existing nature legislation, especially the Habitats and Birds Directives.

**Province of Macerata**

*No. of people interviewed: 1*

The Province of Macerata is one of the five provinces of the Marche region.

The Environment and Water Protection Unit was chosen because of its responsibilities in the implementation of waste water discharge legislation at provincial level.

**Region Emilia-Romagna**

*No. of people interviewed: 2*

Emilia-Romagna is one of the Italian coastal regions of the Adriatic Sea (about 130 km of coasts). Interviews were held with representatives of two Units.

The Unit “Water resources protection and recovery” (1 interviewee) was chosen because it is responsible for the implementation at regional level of the MSFD, the WFD and related legislation.

The Unit “Soil and coastal defence, and remediation” (1 interviewee) was chosen because it is responsible for the management of coastal hazards and for the implementation of the regional Integrated Coastal Zone Management Plan (ICZM).

**Region Marche**

*No. of people interviewed: 7*

Marche is one of the Italian coastal regions of the Adriatic Sea (about 180 Km of coasts). Interviews were held with representatives of the following Units: Fisheries and Hunting; Coastal Defence; Marine Strategy Framework Directive Implementation; Maritime State Property Administration; and Biodiversity, Ecological Networks and Animal Protection.

The Units were chosen because involved in the implementation of marine/maritime legislation at regional level. More into detail:

- Fisheries and Hunting Unit (1 interviewee) – responsible for the implementation of regional fisheries policies;
- Coastal Defence (1 interviewee) – responsible for coastal erosion and hazards, and for the ICZM Plan;
- MSFD (1 interviewee) – responsible for the implementation of the MSFD;
- State Property (1 interviewee) – responsible for the coordination of municipalities in the management of tourism and other coastal activities;
Biodiversity (3 interviewees) – responsible for the management of coastal Natura 2000 sites and other protected areas in the region.

**ARPAM – Marche Region Environmental Protection Agency**

*No. of people interviewed: 2*

The main duties of the ARPAM are, among others: environmental analysis, measures, monitoring and sampling; investigations of environmental risks prevention and control; technical support to Region and Provinces on waste control and management; educational activities and professional training; support to regional Environmental Impact Assessments (EIA).

The ARPAM was chosen because it is responsible for coastal and marine monitoring in the stretch of the Adriatic Sea under jurisdiction of the Marche region.

**CNR ISMAR – Marine Science Institute of the Italian National Research Council**

*No. of people interviewed: 1*

CNR-ISMAR is the section of CNR devoted to marine studies. It conducts research in Mediterranean, oceanic and polar regions, in the fields of oceanography, geology and geophysics, coastal systems and human impacts, climate and paleoclimate, ecosystems and biogeochemistry, and fisheries and aquaculture.

ISMAR was chosen because it is involved in marine data collection and monitoring activities in the Adriatic Sea.

**ISPRA – Italian National Institute for Environmental Protection and Research**

*No. of people interviewed: 1*

ISPRA is the national centre for scientific research in the field of environmental protection. The person interviewed was working at the Water Department, Marine Waters Unit.

The Water Department, Marine Waters Unit was chosen because it provides scientific and technical support to the Ministry of Environment, for the implementation of the MSFD.

**Lega Pesca – Association of Cooperatives operating in the Fisheries Sector**

*No. of people interviewed: 1*

Association of cooperatives of the fisheries sector; it is part of LEGACOOP (National Association of Cooperatives and Mutual Aid Societies), the oldest and biggest Italian cooperative organisation, with more than 13,000 members.

Lega Pesca was chosen because it represents the interests of fishermen, and was expected to provide their point of view on the implementation of existing fisheries and environmental legislation.

**WWF – World Wildlife Fund Italy, Marche Region**

*No. of people interviewed: 1*

The World Wide Fund for Nature is a global organisation for nature conservation. The Italian branch was born in 1966 and is divided into 19 regional sections, with about 200 territorial structures.

The WWF was chosen because of its interests in nature protection and the work done at national level for the protection of the marine environment.

## References

[bib1] Abramic A., Smits P.S., Nunes de Lima V. (2015). Marine Pilot – Analysis of Requirements that Link INSPIRE and MSFD. https://joinup.ec.europa.eu/sites/default/files/ckeditor_files/files/D1-2%20Analysis%20of%20MSFD%20and%20INSPIRE%20requirements%20REV%202.pdf.

[bib2] Arkema K.K., Abramson S.C., Dewsbury B.M. (2006). Marine ecosystem-based management: from characterization to implementation. Front. Ecol. Environ..

[bib3] Barbanti A., Campostrini P., Musco F., Sarretta A., Gissi E. (2015). ADRIPLAN Conclusions and Recommendations: a Short Manual for MSP Implementation in the Adriatic-ionian Region.

[bib4] Barbier E.B. (2012). Progress and challenges in valuing coastal and marine ecosystem services. Rev. Environ. Econ. Policy.

[bib5] Bellas J. (2014). The implementation of the marine strategy framework directive: shortcomings and limitations from the Spanish point of view. Mar. Policy.

[bib6] Berkes F., Colding J., Folke C. (2003). Navigating Social-ecological Systems - Building Resilience for Complexity and Change.

[bib7] Bigagli E. (2015). The European Union legal framework for the management of marine complex social-ecological systems. Mar. Policy.

[bib8] Bigagli E. (2016). The international legal framework for the management of the global oceans social-ecological system. Mar. Policy.

[bib9] Bigagli E. (2017). Marine Complex Adaptive Systems: Theory, Legislation and Management Practices.

[bib10] Binder C.R., Hinkel J., Bots P.W.G., Pahl-Wostl C. (2013). Comparison of frameworks for analyzing social-ecological systems. Ecol. Soc..

[bib11] Blackford J., Jones N., Holt J., Widdicombe S., Lowe D., Rees A. (2009). An initial assessment of the potential environmental impact of CO2 escape from marine carbon capture and storage systems. Proceed. Inst. Mech. Eng. Part A J. Power Energy.

[bib12] Borja A., Elliott M., Andersen J.H., Berg T., Carstensen J., Halpern B.S., Heiskanen A.-S., Korpinen S., Lowndes J.S.S., Martin G., Rodriguez-Ezpeleta N. (2016). Overview of integrative assessment of marine systems: the ecosystem approach in practice. Front. Mar. Sci..

[bib13] Borja A., Elliott M., Snelgrove P.V., Austen M.C., Berg T., Cochrane S., Carstensen J., Roberto D., Greenstreet S., Heiskanen A., Lynam C.P., Mea M., Newton A., Patrício J., Uusitalo L., Uyarra M.C., Wilson C. (2016). Bridging the gap between policy and science in assessing the health status of marine ecosystems. Front. Mar. Sci..

[bib14] Boyes S.J., Elliott M. (2014). Marine legislation - the ultimate “horrendogram”: international law, European directives & national implementation. Mar. Pollut. Bull..

[bib15] Boyes S.J., Elliott M., Murillas-Maza A., Papadopoulou N., Uyarra M.C. (2016). Is existing legislation fit-for-purpose to achieve good environmental <Status in European seas?. Mar. Pollut. Bull..

[bib16] Brennan J., Fitzsimmons C., Gray T., Raggatt L. (2014). EU marine strategy framework directive (MSFD) and marine spatial planning (MSP): which is the more dominant and practicable contributor to maritime policy in the UK?. Mar. Policy.

[bib17] Carpenter S., Walker B., Anderies J.M., Abel N. (2001). From metaphor to measurement: resilience of what to what?. Ecosystems.

[bib18] Cavallo M., Elliott M., Touza J., Quintino V. (2016). The ability of regional coordination and policy integration to produce coherent marine management: implementing the Marine Strategy Framework Directive in the North-East Atlantic. Mar. Policy.

[bib19] Cinnirella S., March D., O'Higgins T., Murciano C., Sardà R., Albaigés J., Pirrone N. (2012). A multidisciplinary spatial data infrastructure for the mediterranean to support the implementation of the marine strategy framework directive. Int. J. Spatial Data Infrastruct. Res..

[bib20] Costanza R. (1999). The ecological, economic and social importance of the oceans. Ecol. Econ..

[bib21] Curtin R., Prellezo R. (2010). Understanding marine ecosystem based management: a literature review. Mar. Policy.

[bib22] De Jonge V.N. (2007). Toward the application of ecological concepts in EU coastal water management. Mar. Pollut. Bull..

[bib23] De Jonge V.N., Elliot M., Brauer V.S. (2006). Marine monitoring: its shortcomings and mismatch with the EU Water Framework Directive's objectives. Mar. Pollut. Bull..

[bib24] De Jonge V.N., Pinto, Turner R.K. (2012). Integrating ecological, economic and social aspects to generate useful management information under the EU Directives' ‘ecosystem approach’. Ocean Coast. Manag..

[bib25] Dimitrakopoulos D., Richardson J., Richardson J. (2001). Implementing EU public policy. European Union. Power and Policy-making.

[bib26] Dryzek J. (2013). The Politics of the Earth: Environmental Discourses.

[bib27] Eason T., Garmestani A.S., Stow C.A., Rojo C., Alvarez-Cobelas M., Cabezas H. (2016). Managing for resilience: an information theory-based approach to assessing ecosystems. J. Appl. Ecol..

[bib28] European Commission (2014). COM(2014) 357 Final – Communication from the Commission to the European Parliament, the Council, the European Economic and Social Committee and the Committee of the Regions Concerning the European Union Strategy for the Adriatic and Ionian Region.

[bib29] European Commission (2017). SWD(2017) 1 Final – COMMISSION STAFF WORKING DOCUMENT Accompanying the Document Report from the Commission to the European Parliament and the Council Assessing Member States' Monitoring Programmes under the Marine Strategy Framework Directive.

[bib30] Fox A.D., Desholm M., Kahlert J., Christensen T.K., Krag Petersen I. (2006). Information needs to support environmental impact assessment of the effects of European marine offshore wind farms on birds. Ibis.

[bib31] Foxon T.J., Reed M.S., Stringer L.C. (2009). Governing long-term Social-Ecological change: what can the Adaptive Management and Transition Management approaches learn from each other?. Environ. Policy Gov..

[bib32] Freire-Gibb L.C., Koss R., Margonski P., Papadopoulou N. (2014). Governance strengths and weaknesses to implement the marine strategy framework directive in European waters. Mar. Policy.

[bib33] Grumbine R.E. (1994). What is ecosystem management?. Conserv. Biol..

[bib34] Guerry A.D. (2005). Icarus and Daedalus: conceptual and tactical lessons for marine ecosystem-based management. Front. Ecol. Environ..

[bib35] Halpern B.S., Walbridge S., Selkoe K.A., Kappel C.V., Micheli F., D'Agrosa C., Bruno J.F., Casey K.S., Ebert C., Fox H.E., Fujita R., Heinemann D., Lenihan H.S., Madin E.M.P., Perry M.T., Selig E.R., Spalding M., Steneck R., Watson R. (2008). A global map of human impact on marine ecosystems. Science.

[bib36] Hattam C., Atkins J.P., Beaumont N., Bӧrger T., Bӧhnke-Henrichs A., Burdon D., de Groot R., Hoefnagel E., Nunes P.A.L.D., Piwowarczyk J., Sastre S., Austen M.C. (2015). Marine ecosystem services: linking indicators to their classification. Ecol. Indic..

[bib37] Hummel H., Frost M., Juanes J., Kochmann J., Castellanos Perez Bolde C., Aneiros F., De Matos V. (2015). A comparison of the degree of implementation of marine biodiversity indicators by European countries in relation to the Marine Strategy Framework Directive (MSFD). J. Mar. Biol. Assoc. U. K..

[bib38] IOC/UNESCO, IMO, FAO, UNDP (2011). A Blueprint for Ocean and Coastal Sustainability.

[bib39] ISPRA (2012). Strategia per l'ambiente marino. La valutazione iniziale dello stato dell'ambiente marino e proposte per la determinazione del buono stato ambientale e la definizione dei traguardi ambientali. http://cdr.eionet.europa.eu/it/eu/msfd8910/msfd4text/envuhgcpg/.

[bib40] Jouanneau C., Raakjaer J. (2014). ‘The hare and the tortoise’: lessons from Baltic sea and mediterranean sea governance. Mar. Policy.

[bib41] Katsanevakis S., Stelzenmueller V., South A., Sorensen T.K., Jones P.J.S., Kerr S., Badalamenti F., Anagnostou C., Breen P., Chust G., D'Anna G., Duijn M., Filatova T., Fiorentino F., Hulsman H., Johnson K., Karageorgis A.P., Kröncke I., Mirto S., Pipitone C., Portelli S., Qiu W., Reiss H., Sakellariou D., Salomidi M., van Hoof L., Vassilopoulou V., Vega Fernández T., Vöge S., Weber A., Zenetos A., ter Hofstede R. (2011). Ecosystem-based marine spatial management: review of concepts, policies, tools, and critical issues. Ocean Coast. Manag..

[bib42] Kern F., Smith A. (2008). Restructuring energy systems for sustainability? Energy transition policy in The Netherlands. Energy Policy.

[bib43] Levin P.S., Fogarty M.J., Murawski S.A., Fluharty D. (2009). Integrated ecosystem assessments: developing the scientific basis for ecosystem-based management of the ocean. PLoS Biol..

[bib44] Milieu (2014). Article 12 Technical Assessment of the MSFD 2012 Obligations – Italy. https://circabc.europa.eu/webdav/CircaBC/env/Marine%20Strategy/Library/MSFDdocuments/A1%20-%20Official%20Documents/MSFD%20Article%2012%20report/MSFD%20Article%2012%20Commission%20assessment%20on%20articles%208-9-10/2014%20-%20Country%20reports/SI%20-%20Slovenia/Art12%20assessment%20-%20SI%20national%20report%20FINAL.pdf.

[bib45] Millard K., Smits P., Abramic A., Calewaert J.B., Shepherd I. (2015). Marine Pilot - EMODnet and INSPIRE: Benefits of Closer Collaboration and a Framework for Action. https://circabc.europa.eu/d/a/workspace/SpacesStore/d949cc14-31aa-49e9-983a-158759f84c25/DIKE_12-2015-05b_EMDODnet_and_INSPIRE.PDF.

[bib46] Olsson P., Galaz V., Boonstra W.J. (2014). Sustainability Transformations: a Resilience Perspective.

[bib47] Palialexis A., Tornero V., Barbone E., Gonzalez D., Hanke G., Cardoso A.C., Hoepffner N., Katsanevakis S., Somma F., Zampoukas N. (2014). In-depth Assessment of the EU Member States' Submissions for the Marine Strategy Framework Directive under Articles 8, 9 and 10.

[bib48] Pereira L., Karpouzoglou T., Doshi S., Frantzeskaki N. (2015). Organising a safe space for navigating social-ecological transformations to sustainability. Int. J. Environ. Res. Public Health.

[bib49] Pinto R., de Jonge V.N., Neto J.M., Domingos T., Marques J.C., Patricio J. (2013). Towards a DPSIR driven integration of ecological value, water uses and ecosystem services for estuarine systems. Ocean Coast. Manag..

[bib50] Ruckelshaus M., Klinger T., Knowlton N., DeMaster D.P. (2008). Marine ecosystem-based management in practice: scientific and governance challenges. Bioscience.

[bib51] Salomon M., Dross M. (2013). Challenges in cross-sectoral marine protection in Europe. Mar. Policy.

[bib52] Smith A., Kern F. (2009). The transitions storyline in Dutch environmental policy. Environ. Polit..

[bib53] Tallis H., Levin P.S., Ruckelshaus M., Lester S.E., McLeod K.L., Fluharty L., Halpern B.S. (2010). The many faces of ecosystem-based management: making the process work today in real places. Mar. Policy.

[bib54] Tunesi L., Casazza G., Dalù M., Giorgi G., Silvestri C. (2013). The implementation of the Marine Strategy Framework Directive in Italy: knowledge to support the management. Biol. Mar. Mediterr..

[bib55] Van der Brugge R., Van Raak R. (2007). Facing the adaptive management challenge: insights from transition management. Ecol. Soc..

[bib56] Van Leeuwen J., van Hoof L., van Tatenhove J. (2012). Institutional ambiguity in implementing the European union marine strategy framework directive. Mar. Policy.

[bib57] Van Tatenhove J., Raakjaer J., van Leeuwen J., van Hoof L. (2014). Regional cooperation for European seas: governance models in support of the implementation of the MSFD. Mar. Policy.

[bib58] Van Tatenhove J.P.M. (2013). How to turn the tide: developing legitimate marine governance arrangements at the level of the regional seas. Ocean Coast. Manag..

[bib59] Van Tatenhove J.P.M. (2016). The environmental state at sea. Environ. Polit..

[bib60] Voss J.P., Bornemann B. (2011). The politics of reflexive governance: challenges for designing adaptive management and transition management. Ecol. Soc..

[bib61] Wing Goodale M., Milman A. (2014). Cumulative adverse effects of offshore wind energy development on wildlife. J. Environ. Plan. Manag..

[bib62] Zampoukas N., Piha H., Bigagli E., Hoepffner N., Hanke G., Cardoso A.C. (2013). Marine monitoring in the European union: how to fulfil the requirements for the marine strategy framework directive in an efficient and integrated way. Mar. Policy.

